# Supplementary material for: In situ strategy for biomedical target localization via nanogold nucleation and secondary growth
Source: Commun Biol. 2021 Jun 10;4:710. doi: 10.1038/s42003-021-02246-3 (PMC8192519; doi:10.1038/s42003-021-02246-3)
Supplement: Supplementary file 4 — Reporting Summary [file 42003_2021_2246_MOESM4_ESM.pdf]

nature research

Corresponding author(s): Akira Sawaguchi

Last updated by author(s): Oct 19, 2020

Reporting Summary

Nature Research wishes to improve the reproducibility of the work that we publish. This form provides structure for consistency and transparency in reporting. For further information on Nature Research policies, see our Editorial Policies and the Editorial Policy Checklist.

Statistics

For all statistical analyses, confirm that the following items are present in the figure legend, table legend, main text, or Methods section.

n/a	Confirmed	
☐	☒	The exact sample size (*n*) for each experimental group/condition, given as a discrete number and unit of measurement
☐	☒	A statement on whether measurements were taken from distinct samples or whether the same sample was measured repeatedly
☐	☒	The statistical test(s) used AND whether they are one- or two-sided<br>*Only common tests should be described solely by name; describe more complex techniques in the Methods section.*
☐	☒	A description of all covariates tested
☐	☒	A description of any assumptions or corrections, such as tests of normality and adjustment for multiple comparisons
☐	☒	A full description of the statistical parameters including central tendency (e.g. means) or other basic estimates (e.g. regression coefficient) AND variation (e.g. standard deviation) or associated estimates of uncertainty (e.g. confidence intervals)
☐	☒	For null hypothesis testing, the test statistic (e.g. *F*, *t*, *r*) with confidence intervals, effect sizes, degrees of freedom and *P* value noted<br>*Give P values as exact values whenever suitable.*
☒	☐	For Bayesian analysis, information on the choice of priors and Markov chain Monte Carlo settings
☒	☐	For hierarchical and complex designs, identification of the appropriate level for tests and full reporting of outcomes
☒	☐	Estimates of effect sizes (e.g. Cohen's *d*, Pearson's *r*), indicating how they were calculated

Our web collection on statistics for biologists contains articles on many of the points above.

Software and code

Policy information about availability of computer code

Data collection	Light microscopic images were collected using Olympus BX51 equipped with a digital camera Olympus DP73.<br>Low-vacuum scanning electron microscopic images were collected using Hitachi-High-Tech TM3030Plus and TM400Plus.<br>Transmission electron microscopic images were collected using Hitachi-High-Tech HT7700.<br>High-resolution scanning Transmission electron microscopic images were collected using Hitachi-High-Tech HD-2700A.<br>All illustrations were drawn using Adobe Photoshop Elements (ver 14.1).
Data analysis	Quantitative analysis of nanogold particles was performed using NIH Image J (ver 1.53c).<br>Statistical analysis was performed using Microsoft Excel (ver 16.4).

For manuscripts utilizing custom algorithms or software that are central to the research but not yet described in published literature, software must be made available to editors and reviewers. We strongly encourage code deposition in a community repository (e.g. GitHub). See the Nature Research guidelines for submitting code & software for further information.

Data

Policy information about availability of data

All manuscripts must include a data availability statement. This statement should provide the following information, where applicable:

- Accession codes, unique identifiers, or web links for publicly available datasets
- A list of figures that have associated raw data
- A description of any restrictions on data availability

The raw data including original light and electron micrographs are available upon request from the corresponding author.

Field-specific reporting

Please select the one below that is the best fit for your research. If you are not sure, read the appropriate sections before making your selection.

☒ Life sciences ☐ Behavioural & social sciences ☐ Ecological, evolutionary & environmental sciences

For a reference copy of the document with all sections, see nature.com/documents/nr-reporting-summary-flat.pdf

Life sciences study design

All studies must disclose on these points even when the disclosure is negative.

Sample size	The numbers of nanogold particles counts (500 particles in each group) were determined based on published studies in the field (ref 26:Leng, W. et al. 2015, ref 27:Kumar, P.S. et al. 2008).
Data exclusions	The average nanogold diameter of the "PBS pretreatment/12 hours incubation" group was excluded because of the emergence of non-specific particles irrelevant to immunoenzymatic DAB products.
Replication	The experimental findings were reliably reproduced at least 3 times for representative data used for statistical analysis. The numbers of nanogold particles counts (500 particles in each group) are described in corresponding figure legends.
Randomization	For the quantification of nanogold sizes, the imaging fields and 500 particles in each groups were randomly selected.
Blinding	Blinding was not relevant to this study because there was no group allocation.

Reporting for specific materials, systems and methods

We require information from authors about some types of materials, experimental systems and methods used in many studies. Here, indicate whether each material, system or method listed is relevant to your study. If you are not sure if a list item applies to your research, read the appropriate section before selecting a response.

Materials & experimental systems

n/a	Involved in the study	
☐	☒	Antibodies
☒	☐	Eukaryotic cell lines
☒	☐	Palaeontology and archaeology
☐	☒	Animals and other organisms
☒	☐	Human research participants
☒	☐	Clinical data
☒	☐	Dual use research of concern

Methods

n/a	Involved in the study	
☒	☐	ChIP-seq
☒	☐	Flow cytometry
☒	☐	MRI-based neuroimaging

Antibodies

Antibodies used	Anti-synaptopodin (clone G1D4; Progen Biotechnik, Heidelberg, Germany)<br>Anti-H+/K+-ATPase (2B6; MBL, Nagoya, Japan)<br>Anti-platelet glycoprotein IIb/IIIa (Affinity Biologicals Inc., Hamilton, CA, USA)
Validation	All primary antibodies are commercially available and validated by the manufacturers, as informed in the following manufacturers' websites.<br><br>Mouse monoclonal antibody against synaptopodin (clone G1D4; Progen Biotechnik, Heidelberg, Germany)<br>https://www.progen.com/ProductLeaflet/file/getpdf/name/anti-SynaptopodinSYNPO_mouse_monoclonal%2C_G1D4%2C_supernatant.pdf?fileId=484

	Mouse monoclonal antibody against H+/K+-ATPase (2B6; MBL, Nagoya, Japan)<br>https://ruo.mbl.co.jp/bio/dtl/dtlfiles/D032-3H_v4.pdf<br><br>Sheep polyclonal antibody against platelet glycoprotein IIb/IIIa (Affinity Biologicals Inc., Hamilton, CA, USA)<br>https://affinitybiologicals.com/product/glycoprotein-2b-3a-polyclonal-antibody/

Animals and other organisms

Policy information about studies involving animals; ARRIVE guidelines recommended for reporting animal research

Laboratory animals	Male Wistar rats (8-10 weeks)<br>Male Japanese white rabbits (weighing 2.5–3.0 kg)<br>Female C57BL/6J mice (8-12 weeks)
Wild animals	The study did not involve wild animals.
Field-collected samples	The study did not involve samples collected from the field.
Ethics oversight	All animal procedures were carried out under protocols approved by the University of Miyazaki Animal Research Committee (#2005-009-1, #2016-513-3, #2016-509-5, #2018-509-1), in accordance with international guiding principles for biomedical research involving animals.

Note that full information on the approval of the study protocol must also be provided in the manuscript.
